# The Effect of the Repression of Oxidative Stress on Tenocyte Differentiation: A Preliminary Study of a Rat Cell Model Using a Novel Differential Tensile Strain Bioreactor

**DOI:** 10.3390/ijms20143437

**Published:** 2019-07-12

**Authors:** Ming-Yen Hsiao, Ping-Cheng Lin, Wei-Hao Liao, Wen-Shiang Chen, Chia-Hsien Hsu, Cheng-Kun He, Ya-Wen Wu, Amit Gefen, Michele Iafisco, Lixin Liu, Feng-Huei Lin

**Affiliations:** 1 Department of Biomedical Engineering, National Taiwan University, Taipei 10617, Taiwan; 2 Departments of Physical Medicine and Rehabilitation, National Taiwan University Hospital, College of Medicine, National Taiwan University, Taipei 10048, Taiwan; 3 Institute of Biomedical Engineering and Nanomedicine, National Health Research Institutes, Miaoli 35053, Taiwan; 4 Ph.D. Program in Tissue Engineering and Regenerative Medicine, National Chung Hsing University, Taichung 40227, Taiwan; 5 Department of Biomedical Engineering, Tel Aviv University, Tel Aviv 61999, Israel; 6 Institute of Science and Technology for Ceramics, 64-48018 Faenza, Italy; 7 School of Materials Science and Engineering, Center of Functional Biomaterials, Key Laboratory of Polymeric Composite Materials and Functional Materials of Education, GD Research Center for Functional Biomaterials Engineering and Technology, Sun Yat-sen University, Guangzhou 510275, China; 8 Director, Institute of Biomedical Engineering and Nanomedicine, National Health Research Institutes, Miaoli 35053, Taiwan

**Keywords:** tendinopathy, oxidative stress, cell model

## Abstract

Because of limitations in the current understanding of the exact pathogenesis of tendinopathy, and the lack of an optimal experimental model, effective therapy for the disease is currently unavailable. This study aims to prove that repression of oxidative stress modulates the differentiation of tendon-derived cells (TDCs) sustaining excessive tensile strains, and proposes a novel bioreactor capable of applying differential tensile strains to cultured cells simultaneously. TDCs, including tendon-derived stem cells, tenoblasts, tenocytes, and fibroblasts, were isolated from the patellar tendons of Sprague‒Dawley rats. Cyclic uniaxial stretching with 4% or 8% strain at 0.5 Hz for 8 h was applied to TDCs. TDCs subjected to 8% strain were treated with epigallocatechin gallate (EGCG), piracetam, or no medication. Genes representing non-tenocyte lineage (*Pparg*, *Sox9*, and *Runx2*) and type I and type III collagen were analyzed by quantitative polymerase chain reaction. The 8% strain group showed increased expression of non-tenocyte lineage genes and type III/type I collagen ratios compared with the control and 4% strain groups, and the increased expression was ameliorated with addition of EGCG and piracetam. The model developed in this work could be applied to future research on the pathophysiology of tendinopathy and development of treatment options for the disease. Repression of oxidative stress diminishes the expression of genes indicating aberrant differentiation in a rat cell model, which indicates potential therapeutic intervention of tendinopathy, the often relentlessly degenerate condition.

## 1. Introduction

Tendinopathy accounts for nearly one-third of all cases of musculoskeletal disorders, exerting great impacts on a patient’s quality of life [1,2]. Over the last several decades, tendinopathy therapy has seen limited progress and current treatments have mostly aimed to provide symptomatic relief [1]. One of the main reasons is that its exact pathogenesis remains incompletely understood, but can include repetitive injury, oxidative stress, ischemia, impaired metabolism, chronic inflammation, and impaired healing [1,2,3,4]. Accumulated evidence indicates the important role of oxidative stress [5,6,7]. Reactive oxygen species (ROS) modulate a variety of physiological events, including cell proliferation and differentiation, inflammation, and healing [8]. Extensive ROS exposure and/or insufficient cellular antioxidant capacity may cause cell injury, senescence, and even death [9].

As a proof of concept, we aim to mitigate the aberrant tenocyte differentiation induced by excessive tensile strain by repression of oxidative stress. Our study is an extension and modification of previous works on the modulation of tendon stem cells’ differentiation by cyclic tensile strain [10,11,12]. Using a similar in vitro mechanical loading system, it was found that excessive strain increases the expression of genes related to chondrogenic, osteogenic, and adipogenic lineages, which is indicative of tendinopathy changes [10,11,12]. By contrast, a small amount of strain promoted TDC differentiation into tenocytes with a fairly regular alignment of cells and ECM [12,13,14].

Epigallocatechin gallate (EGCG) is a natural polyphenol that demonstrates a wide range of cell-protective effects, believed to be mediated by its antioxidant and anti-inflammatory properties [15,16]. Piracetam is a neuroprotective medication effective in improving cognitive impairment. Although the mechanism of this drug is not yet fully understood, it has been found to reduce oxidative stress and inflammatory cytokine production [17,18,19]. These medications have not been used to treat tendinopathy despite previous studies showing their positive effects on various cell types. Therefore, we hypothesize that EGCG and piracetam protect tenocytes from excessive mechanical loading and inhibit non-tenocyte lineage differentiation.

Meanwhile, we developed a novel bioreactor that can exert different ranges of tensile strain on cultured cells simultaneously. The dynamic culture devices used previously can only apply a certain strain to one group at a time, which means that at least two distinct sets of experiments are required to study cellular responses to different degrees of strain. The proposed design enables the simulation of both physiological and pathological conditions in the same set of experiment, and ensures identical experimental conditions except for the degree of strain. 

## 2. Results

### 2.1. Characteristics of Tendon-Derived Cells (TDCs)

**Figure 1:**
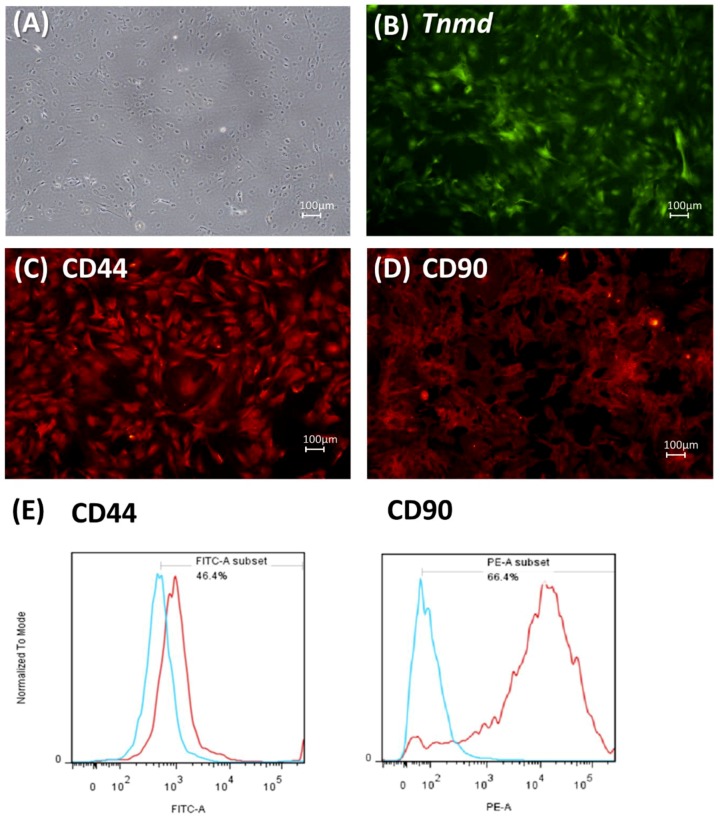
Characteristics of tendon-derived cells (TDCs). Representative photomicrographs (10×) of TDCs after extraction and 7 d of culture (**A**). Immunohistochemistry of tenomodulin (*Tnmd*) (**B**), CD44 (**C**), and CD90 (**D**). (**E**) Flow cytometry assay of the TDCs. The cells expressed CD 44 and CD 90, which are surface markers representing mesenchymal stem cells. Red lines: TDCs stained with CD 44 or CD 90; blue lines: TDCs stained with isotype-matched IgG1 as a negative control.

TDCs from passage 1 to passage 5 (P1–P5) showed elongated shapes resembling fibroblasts and adhered to the culture plate (Figure 1). These cells also showed CD 44 and CD 90 expression. Immunohistochemistry confirmed the expression of tenomodulin (*Tnmd*) in most TDCs.

### 2.2. Effect of Cyclic Uniaxial Stretching on the Gene Expression of TDCs

**Figure 2:**
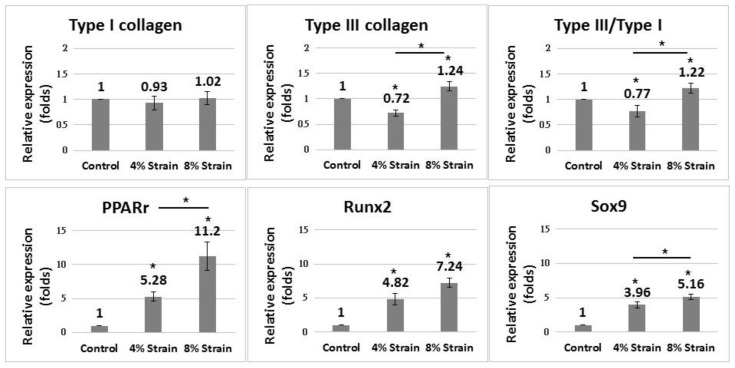
Quantitative PCR of tendon-derived cells (TDCs) 8 h after cyclic stretching with 4% or 8% mean strain. **Upper panel**: The 8% strain group showed increased gene expression of type III collagen and type III/type I collagen ratio compared with cells in the control group; the 4% strain group showed decreased gene expression of type III collagen compared with TDCs in the control group. **Lower panel**: Both the 4% and 8% strain groups showed increased expression of *Pparg*, *Runx2*, and *Sox9* compared with TDCs in the control group. Data are expressed in fold-changes of expression level relative to the control (static culture) and presented as mean ± SD (*n* = 4, * *p* < 0.05).

Compared with the control group, cells subjected to a principal strain of 4% and 8% (referred to as the 4% strain and 8% strain groups, respectively, in the following context) showed increased gene expression of non-tenocyte lineages (*p* < 0.01 in both groups) (Figure 2). The 8% strain group showed more significant increases in the peroxisome proliferator activated receptor gamma (*Pparg*) and *Sox9* than the 4% strain group (*p* < 0.05 in both groups), and differences in *Runx2* between the groups were not significant (*p* = 0.052). While the gene expression of type I collagen did not differ between the groups, the gene expression of type III collagen and ratio of type III/type I collagen increased in the 8% strain group (both *p* < 0.01) but decreased in the 4% strain group (*p* < 0.05 and *p* < 0.01, respectively) compared with the control group.

### 2.3. Effect of EGCG and Piracetam on the Gene Expression of TDCs Subjected to 8% Uniaxial Cyclic Stretching

**Figure 3:**
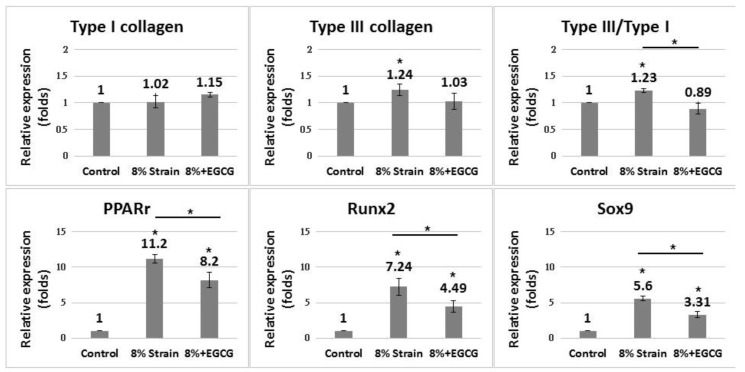
Effect of EGCG on the gene expression of tendon-derived cells (TDCs) subjected to cyclic stretching with 8% strain. **Upper panel**: Quantitative PCR demonstrated that TDCs with EGCG showed decreased expression of type III/type I collagen genes compared with cells without EGCG. **Lower panel**: TDCs with EGCG showed decreased expression of *Pparg*, *Runx2*, and *Sox9* compared with cells without EGCG. Data are expressed in fold-changes of expression level relative to the control (static culture) presented as mean ± SD (*n* = 4, * *p* < 0.05).

EGCG significantly suppressed the increased expression of non-tenocyte lineage genes in the 8% strain group compared with the reference group (*p* < 0.01 in *Pparg* and *Runx2*; *p* < 0.05 in *Sox9*) (Figure 3). EGCG did not significantly alter the expression of type I and type III collagen, but decreased the ratio of type III/type I collagen (*p* < 0.05).

**Figure 4:**
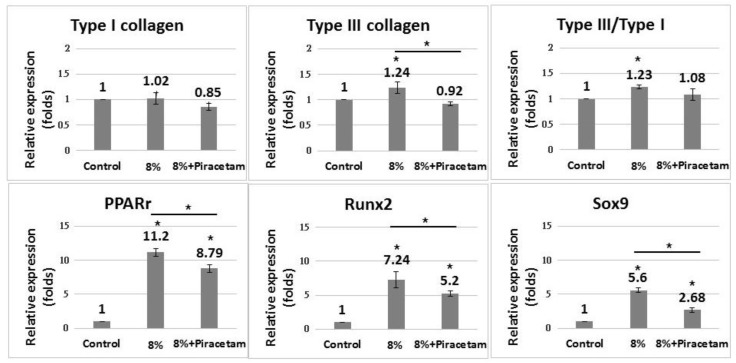
Effect of piracetam on the gene expression of tendon-derived cells (TDCs) subjected to cyclic stretching with 8% strain. **Upper panel**: Quantitative PCR demonstrated that TDCs with piracetam showed decreased expression of type III collagen genes compared with cells without piracetam. The difference in type III/type I ratio between groups was not significant. **Lower panel**: TDCs with piracetam showed decreased expression of *Pparg*, *Runx2*, and *Sox9* compared with cells without piracetam. Data are expressed in fold-changes of expression level relative to the control (static culture) and presented as mean ± SD (*n* = 4, * *p* < 0.05).

The addition of piracetam significantly suppressed the expression of non-tenocyte lineage genes in the 8% strain group compared with the reference group (*p* < 0.05 in *Pparg* and *Runx2*; *p* < 0.01 in *Sox9*) (Figure 4). Piracetam significantly decreased the expression of type III collagen but did not significantly change the type III/type I collagen ratio (*p* = 0.06).

### 2.4. Differential Uniaxial Stretch Culture Device

**Figure 5:**
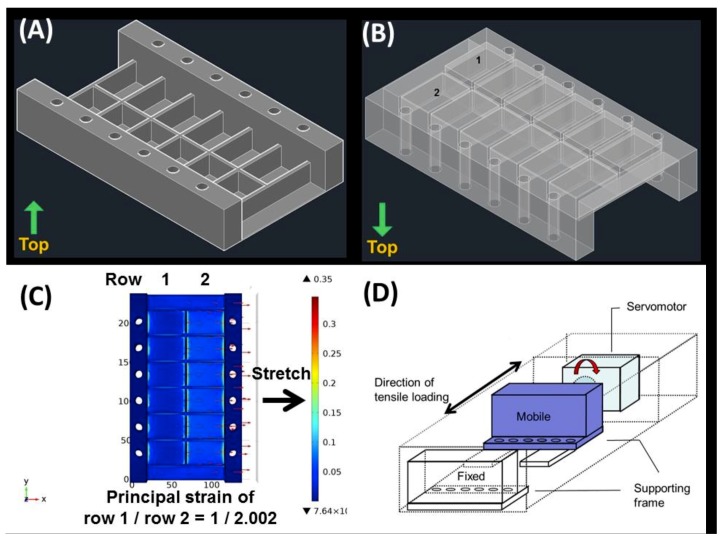
The differential uniaxial stretching device used in this work. (**A**) The elastic culture plate was made of polydimethylsiloxane (PDMS) and partitioned into two rows of chambers. The plate was fixed by pins through vertical holes at the side walls. (**B**) The chambers (1,2) were designed to have bottoms with different thicknesses. (**C**) Upon uniaxial stretching (direction indicated by arrows), the mean strain ratio of row 1 to row 2 was 1:2.002. (**D**) The culture plate was fixed to a supporting frame. One side of the supporting frame was mobile and operated by a servomotor to achieve cyclic uniaxial stretching.

Figure 5 demonstrates the bioreactor constituting a specially designed elastic culture plates made of polydimethylsiloxane (PDMS), a mobile supporting frame, a servomotor, and a control panel. The finite element analysis (COMSOL Multiphysics, District Avenue Burlington, MA, USA) showed that, with a bottom thickness of 6.25 mm for row 1 and 1.0 mm for row 2 of the PDMS culture plate, the first principal strain of the two rows is 3.88% and 7.77%, respectively (at a ratio of 1:2.002). 

## 3. Discussion

Our study showed that the anti-oxidative medications EGCG and piracetam could diminish the expression of genes indicating non-tenocyte lineage differentiation in a rat cell model of tendinopathy induced by cyclic uniaxial stretching. The results indicate that eliminating oxidative stress could be a potential strategy in the treatment of tendinopathy. The novel bioreactor used in the present study is capable of applying different ranges of tensile strains to the culture device simultaneously, and can simulate both physiological and mechanically overloaded conditions, serving as a useful tool in research on the pathophysiology of tendinopathy and the exploration of new therapeutic targets of the disease. 

Given its antioxidative properties, EGCG has presented pleiotropic cell protection effects, including anticancer, cardiovascular protection, and antineurodegenerative activities, in in vitro and animal studies [15,16,20]. In musculoskeletal medicine, a recent study also found that EGCG could inhibit cell death related to ROS in a human intervertebral disk cell model [21]. The mechanism of tendinopathy, which tends to present as accumulated microtrauma, may be similar to that of intervertebral disk degeneration and many other degenerative musculoskeletal conditions. In the present study, EGCG decreased the expression of genes indicating aberrant differentiation of TDCs into non-tenocytes, which is believed to be a key feature of tendinopathy, in a rat cell model.

Piracetam is a nootropic medication widely used to treat cognitive impairment [19]. While the exact mechanism of its therapeutic effect remains unknown, piracetam has been demonstrated by in vitro and in vivo animal studies to be able to attenuate oxidative stress and inflammation [17,18,22,23,24]. The drug ameliorates ischemia‒reperfusion injury in various types of cells [23,24,25]. We thus hypothesize that piracetam could exert positive effects on degenerative musculoskeletal conditions such as tendinopathy even if its application in the musculoskeletal field is limited. Our results showed that the drug could decrease the expression of genes indicating aberrant differentiation of TDCs. 

Besides reducing oxidative injuries, piracetam can facilitate microcirculation at the vascular level. An animal study has found that piracetam could exert analgesic effects by reducing cytokine production in a mouse model [17]. These results indicate that piracetam has promise as a potential medication in future in vivo experiments on tendinopathy treatment. 

After the application of 8% cyclic tensile strain, the TDCs showed increased expression of *Pparg*, *Sox9*, and *Runx2*, indicating non-tenocyte lineage differentiation. These results are compatible with a previous cell model of tendinopathy [11,12]. The device applied in previous tendinopathy studies applies a certain strain [10,11,12]. When studying cellular responses to different strain ratios, two sets of experiments must be performed. There is substantial variation among different groups of cells, such as different passages of cell culture and cells from different subjects. The present study utilizes a bioreactor capable of applying various strain ratios simultaneously to simulate tenocytes in both physiological and pathological conditions. This design enables the direct comparison of cells sustaining different mechanical loads and ensures otherwise identical experimental conditions. 

Previous studies have utilized CD44 and CD90 to identify TDCs [11]. While a flow cytometry assay showed similar results in this study, these markers are actually expressed in mesenchymal stem cells and not exclusively expressed in TDCs [11,26]. Immunohistochemistry of *Tnmd* further confirmed that the TDCs were mostly of tenocyte lineage. *Pparg*, *Sox9*, and *Runx2* were analyzed in order to relate our findings to the existing literature [11,12]. It should be noted that the expression of these genes indicates abnormal TDCs differentiation, but not the differentiation of a certain lineage. Other specific markers should be used in future studies. For example, type II collagen, Aggrecan, and Biglycan could be used as indicators of chondrogenic differentiation, and Scleraxis, Tenascin, and Tenomodulin could be used to confirm tenogenic differentiation [27]. Histologically, tendinopathy produces disorganized fibrillary structures of collagen and increases type III/type I collagen ratios, glycosaminoglycan deposition [28,29], and aberrant differentiation of TDCs into non-tenocytes (tissue metaplasia) [30,31,32]. Thus, in the present study, the genes of type III and type I collagen, as well as *Pparg*, *Sox9*, and *Runx2*, were used as indicators of non-tenogenic differentiation in TDCs. 

This study has several limitations. 

First, the TDCs isolated in this study include a miscellaneous collection of cells, including tendon-derived stem cells, tenoblasts, tenocytes, and fibroblasts. The presence of specific cells could not be confirmed because of the lack of specific markers for each type of cells. Of note, in physiological conditions the tissue response to external stimuli is often the collective results of complex interactions between different cells. We believe that metaplasia, which is the aberrant differentiation of tissues, cannot be attributed to a single cell type, and that the use of a miscellaneous collection of cells did not significantly influence our results. 

Second, the experiment was performed in 2D culture condition and the cells were mechanically loaded without interaction with sufficient ECM, which is fairly different from actual 3D physiological conditions. In the present study, the effective strain cultured cells actually received was not confirmed and the result cannot be extrapolating to in vivo condition. Nonetheless, the model applied was modified from a previous experiment that had successfully induced non-tenogenic differentiation after mechanical loading with a large strain [10,11,12]. To minimize errors, non-adherent cells were washed away before the application of cyclic stretching. 

Third, the present study analyzed gene expression at the cellular level after 8 h of cyclic stretching, and end-product and histopathological analyses were not performed. The stretching device would require further modification to allow these analyses because these operations require much longer culture times and a change in medium. Further ex vivo and in vivo studies would provide more solid evidence of the therapeutic effect of the medications studied in this work. Finally, mechanical overloading only represents one component of the complex etiology of tendinopathy. The effects of other factors, such as cytokines and oxidative stress, require further elucidation. Research on the pathophysiology and possible treatment of tendinopathy may necessitate multiple models to represent different stages and conditions of the disease.

## 4. Materials and Methods

This study follows the National Institutes of Health Guide for the Care and Use of Laboratory Animals (NIH Publication No. 8023, revised in 1978). Experiments were conducted in accordance with Institution Guidelines and were approved under the Affidavit of Approval of Animal Use Protocol, Institutional Animal Care and Use Committee (IACUC), College of Medicine and College of Public Health, National Taiwan University (IACUC Approval No: 20150375, Aug. 1, 2016 to Jul. 31, 2019).

### 4.1. Study Protocol

TDCs were isolated from the patellar tendons of Sprague‒Dawley rats (anesthetized on the day of arrival) and cultured for seven days according to previously described protocols [10,11,12]. Note that TDCs include a miscellaneous collection of cells, including tendon-derived stem cells, tenoblasts, tenocytes, and fibroblasts. CD44 and CD90, which are the surface molecules of mesenchymal stem cells, were used as surrogate markers for TDC identification by flow cytometry assay [11], because of the lack of a specific tendon cell marker.

The isolated TDCs were seeded on the culture plates at a density of 10^4^ cells/cm^2^. Non-adherent cells were removed by washing with PBS 24 h after culture. After another 24 h, the growth medium was replaced, and cyclic uniaxial stretching was applied at a frequency of 0.5 Hz for 8 h so that the strain ratios of the two rows of cultural plates reached 4% and 8%. Cells in culture plates without stretching served as the reference group. 

Cells subjected to 8% strain were further divided into two groups. In the experimental group, 2 mL of 5 μM EGCG or 0.1 mM piracetam (Sigma-Aldrich, St. Louis, MO, USA) were added to the cells before the start of cyclic stretching. Cells without medication served as the reference group. 

After mechanical loading, the cells were collected, and their RNA was extracted and analyzed by quantitative polymerase chain reaction (PCR) for type I collagen, type III collagen, and *Pparg*, *Sox-9*, and *Runx2*, which are non-tenocyte lineage genes [11,12]. TDCs without mechanical loading served as the control group.

TDCs from both patellar tendons of four male Sprague‒Dawley rats were used in each experiment. Each of the following experiments were performed in triplicate. The experiment protocol is described in detail in the following sections.

### 4.2. Differential Uniaxial Stretch Culture Device

To simulate both physiological and pathological conditions in one experiment, we developed a novel bioreactor featuring specially designed elastic culture plates made of PDMS, which achieve different degrees of tensile strain upon uniaxial mechanical stretching. First, a 3D model of the culture plates was designed using AutoCAD software (2014 SP1, Autodesk, Inc., San Rafael, CA, USA). The culture plate was partitioned into two rows of chambers with bottoms featuring different thicknesses. Then the 3D model was entered into the COMSOL Multiphysics software (District Avenue Burlington, MA, USA) and finite element analysis was performed by setting the model material and boundary conditions to simulate the strain ratio after stretching. The density of PDMS is 970 kg/m^3^ and the Young’s modulus of PDMS is 750 kPa. The target strain ratio of the two rows of chambers is 1:2 upon uniaxial stretching (Figure 5).

During cyclic stretching, a total strain of 12% was applied to the PDMS plate so that the mean strain of the two rows was 4% and 8%, respectively. The bioreactor accomplished cyclic uniaxial stretching via a servomotor with adjustable displacement and frequency. 

The culture plate was pretreated with a gelatin solution. In brief, gelatin powder (Sigma-Aldrich) was dissolved in double-distilled H2O to 2% (*w*/*v*). The solution was heated to 37 °C for 10 min and then passed through a 0.22-μm filter. Two milliliters of gelatin solution were added to each well of a PDMS plate placed in a 37 °C incubator for 2 h.

### 4.3. Isolation and Culture of TDCs

Rat TDCs were isolated according to previously described protocols [10,11,12]. The patellar tendons of both limbs were harvested from four male Sprague‒Dawley rats aged 4–6 weeks and weighing about 250–300 g; these rats were euthanized with isofluorane (300–600 mL/min) prior to sample collection. The middle portion of the patellar tendon, which was about 1 mm below the patella and 1 mm above the tibial insertion, was excised. After removal of the adjacent connective tissues, the tendons were minced, digested with type I collagenase (3 mg/mL; Sigma-Aldrich) for 2.5 h, and then passed through a 70 mm BD Falcon™ cell strainer (BD Biosciences, San Diego, CA, USA) to yield a single-cell suspension. The cells were then centrifuged at 300× *g* for 5 min, washed with sterile phosphate-buffered saline (PBS), and resuspended in Dulbecco’s Modified Eagle Medium, 10% fetal bovine serum (FBS), 100 U/mL penicillin, and 100 mg/mL streptomycin, all of which were obtained from Sigma-Aldrich. The isolated cells were cultured at 37 °C with 5% CO_2_ at a cell density of 10^4^ cells/cm^2^. Non-adherent cells were removed by washing with PBS 24 h after culture. On day 7, the cells were trypsinized and mixed together as passage 0 (P0). Cells from P1–P5 were used for all experiments, and the medium was changed every three days.

### 4.4. Flow Cytometry Assay

Phycoerythrin (PE) or fluorescein isothiocyanate (FITC) (BD Biosciences)-conjugated mouse anti-rat monoclonal anti-CD44 (550974; BD Biosciences) and anti-CD90 (551401; BD Biosciences) were used. PE- or FITC-conjugated isotype-matched IgG1 were used as negative controls (IC002P or IC002F, R&D systems, Inc., Minneapolis, US). TDCs (5 × 10^5^; P1) were incubated with 1 μg of antibodies away from the light for 45 min at room temperature. After washing and centrifugation at 4000× *g* for 5 min, the cells were resuspended in 300 μL of ice cold PBS (with 10% FBS and 1% sodium azide) and subjected to fluorescence-activated cell sorting (FACS) analysis (BD Biosciences). A BD FACSCalibur™ system (BD Biosciences) was used to calculate the percentage of cells with positive signals. 

### 4.5. Immunohistochemistry

TDCs were seeded at a cell density of 5 × 10^4^ cells/well and incubated at 37 °C at 5% CO_2_. After 24 h incubation, cells were fixed at 37 °C for 10 min with 4% formaldehyde (pH 7.4). They were then washed twice with PBS and permeabilized at room temperature for 10 min. Next, cells were blocked in blocking solution (2% bovine serum albumin in PBS) for 1 h at room temperature, incubated overnight with primary antibodies diluted in blocking solution at 4 °C, washed three times for 5 min with PBS, incubated with secondary antibody at RT for 2 h, and washed four times for 5 min with PBS. The primary antibodies used for immunostaining and their dilutions were as follows: rabbit anti-tenomodulin (1:50, Abcam, Cambridge, UK), mouse anti-CD44 (1:50, BD Biosciences), mouse anti-90(1:50, BD Biosciences). The secondary antibodies used were Alexa Fluor 488-conjugated goat anti-mouse IgG (1:100, Thermo, Waltham, US) and Alexa Fluor 555-conjugated goat anti-rabbit IgG (1:100, Thermo).

### 4.6. Quantitative PCR

Quantitative PCR was performed using a Step-One Plus Real-Time PCR System (Applied Biosystems, Foster City, CA, USA) according to the manufacturer’s instructions with FastStart Universal SYBR Green Master (Rox) reagent. The primer sequences used in this study were as follows: β-actin: forward 5′-ATCGTGGGCCGCCCTAGGCA-3′, reverse 5′-TGGCCTTAGGGTTCAGAGGGG-3′; Type I collagen: forward 5′-ATCCTGCCGATGTCGCTAT-3′, reverse 5′-CCACAAGCGTGCTGTAGGT-3′; Type III collagen: forward 5′-CTGGTCCTGTTGGTCCATCT-3′, reverse 5′-ACCTTTGTCACCTCGTGGAC-3′; Pparg: forward 5′-CTGACCCAATGGTTGCTGATTAC-3′, reverse 5′-GGACGCAGGCTCTACTTTGATC-3′; *Runx2*: forward 5′-CCGATGGGACCGTGGTT-3′, reverse 5′-CAGCAGAGGCATTTCGTAGCT-3′; and Sox-9: forward 5′- CTGAACGAGAGCGAGAAG-3′, reverse 5′-TTCTTCACCGACTTCCTCC-3′. Cytoplasmic RNA was extracted using an RNeasy^®^ Mini Kit and then reverse-transcribed to complementary DNA by the SuperScript III First-Strand Synthesis System. 

The PCR conditions were as follows: denaturation at 50 °C for 2 min and 95 °C for 10 min and 40 cycles at 95 °C for 15 s and 58–60 °C for 60 s. β-actin was used as the internal control. Data were analyzed using the 2−ΔΔCt method and expressed as fold-changes compared with the non-loading group.

### 4.7. Statistical Analysis

The data were presented as mean standard deviation (SD). Comparisons between groups were performed by ANOVA, followed by post hoc Tukey’s tests. All analyses were done using SPSS version 16.0 software (SPSS Inc., Chicago, IL, USA). Statistical significance was set at *p* < 0.05.

## 5. Conclusions

EGCG and piracetam reduced non-tenocyte gene expression in TDCs sustaining excessive strain, indicating that eliminating oxidative stress could be a potential strategy in the treatment of tendinopathy. The model could be applied in future research on the pathophysiology and treatment of tendinopathy. 

## Abbreviations

EGCG	epigallocatechin gallate
TDCs	tendon-derived cells
ECM	extracellular matrix
ROS	reactive oxygen species
PCR	polymerase chain reaction
PBS	phosphate-buffered saline
FBS	fetal bovine serum
PDMS	polydimethylsiloxane
PE	phycoerythrin
FITC	fluorescein isothiocyanate
FACS	fluorescence-activated cell sorting
